# A Novel Cold-Adapted Leucine Dehydrogenase from Antarctic Sea-Ice Bacterium *Pseudoalteromonas* sp. ANT178

**DOI:** 10.3390/md16100359

**Published:** 2018-10-01

**Authors:** Yatong Wang, Yanhua Hou, Yifan Wang, Lu Zheng, Xianlei Xu, Kang Pan, Rongqi Li, Quanfu Wang

**Affiliations:** School of Marine Science and Technology, Harbin Institute of Technology, Weihai 264209, China; wangyatong199311@163.com (Y.W.); marry7718@163.com (Y.H.); daid01@126.com (Y.W.); zhenglu0206@126.com (L.Z.); 17863108956@163.com (X.X.); m15662319228@163.com (K.P.); l1263769417@163.com (R.L.)

**Keywords:** leucine dehydrogenase, cold-adapted, Antarctic bacterium, sea-ice, homology modeling

## Abstract

l-*tert*-leucine and its derivatives are useful as pharmaceutical active ingredients, in which leucine dehydrogenase (LeuDH) is the key enzyme in their enzymatic conversions. In the present study, a novel cold-adapted LeuDH, *psleudh*, was cloned from psychrotrophic bacteria *Pseudoalteromonas* sp. ANT178, which was isolated from Antarctic sea-ice. Bioinformatics analysis of the gene *psleudh* showed that the gene was 1209 bp in length and coded for a 42.6 kDa protein containing 402 amino acids. PsLeuDH had conserved Phe binding site and NAD^+^ binding site, and belonged to a member of the Glu/Leu/Phe/Val dehydrogenase family. Homology modeling analysis results suggested that PsLeuDH exhibited more glycine residues, reduced proline residues, and arginine residues, which might be responsible for its catalytic efficiency at low temperature. The recombinant PsLeuDH (rPsLeuDH) was purified a major band with the high specific activity of 275.13 U/mg using a Ni-NTA affinity chromatography. The optimum temperature and pH for rPsLeuDH activity were 30 °C and pH 9.0, respectively. Importantly, rPsLeuDH retained at least 40% of its maximum activity even at 0 °C. Moreover, the activity of rPsLeuDH was the highest in the presence of 2.0 M NaCl. Substrate specificity and kinetic studies of rPsLeuDH demonstrated that l-leucine was the most suitable substrate, and the catalytic activity at low temperatures was ensured by maintaining a high *k*_cat_ value. The results of the current study would provide insight into Antarctic sea-ice bacterium LeuDH, and the unique properties of rPsLeuDH make it a promising candidate as a biocatalyst in medical and pharmaceutical industries.

## 1. Introduction

Leucine dehydrogenase (LeuDH; EC 1.4.1.9), a NAD^+^ dependent oxidoreductase, which catalyzes reversible l-leucine and other branched chain l-amino acids deamination reaction to the formation of the corresponding α-keto acid [1]. The enzyme was first identified in *Bacillus cereus* [2], and then was found in some microorganisms *Bacillus licheniformis* [3], *Bacillus sphaericus* [4], *Citrobacter freundii* [5], and *Laceyella sacchari* [6]. Moreover, crystal structures of the LeuDH from *Sporosarcina psychrophila* [7] and *Bacillus sphaericus* have been described [8].

LeuDH is used as a biocatalyst to format amino acids for using in the pharmaceutical industry by catalyzing the corresponding α-keto acids [9]. However, some of α-keto acids are unstable and degraded during prolonged incubation at moderate temperatures, such as 37 °C [10]. Importantly, cold-adapted enzymes that exhibit high levels of activity at room temperature (20–25 °C) should be useful for converting such unstable α-keto acids. What is more, cold-adapted enzymes have better conversion rates, the specificity of substrate and product, fewer by-products, which are required in the modern industry [11]. Although many LeuDHs have already been characterized, only a few cold-adapted LeuDH have been reported, such as LeuDH from *Alcanivorax dieselolei* [12] and *Sporosarcina psychrophila* [7].

Antarctic sea-ice, due to its specific geographical location and climate, is considered as an extreme environment on the earth. To develop the ability to withstand the extreme environment, sea-ice microorganisms have evolved several adaptive strategies and would be the new and promising microbial sources of cold-adapted enzymes. In our previous studies, some cold-adapted enzymes were isolated from Antarctic sea-ice bacteria and had become interesting for industrial applications [13,14]. It is well-known that l-*tert*-leucine and its derivatives are useful as pharmaceutical active ingredients and chiral auxiliaries, while LeuDH is a key enzyme for the enzymatic production of l-*tert*-leucine. Here, we briefly describe the homology modeling, expression, and characterization of cold-adapted LeuDH from Antarctic sea-ice bacterium. This LeuDH had unique properties make it good candidate for future medical and pharmaceutical industry applications.

## 2. Results and Discussion

### 2.1. Gene Cloning and Sequence Analysis

**Figure 1:**
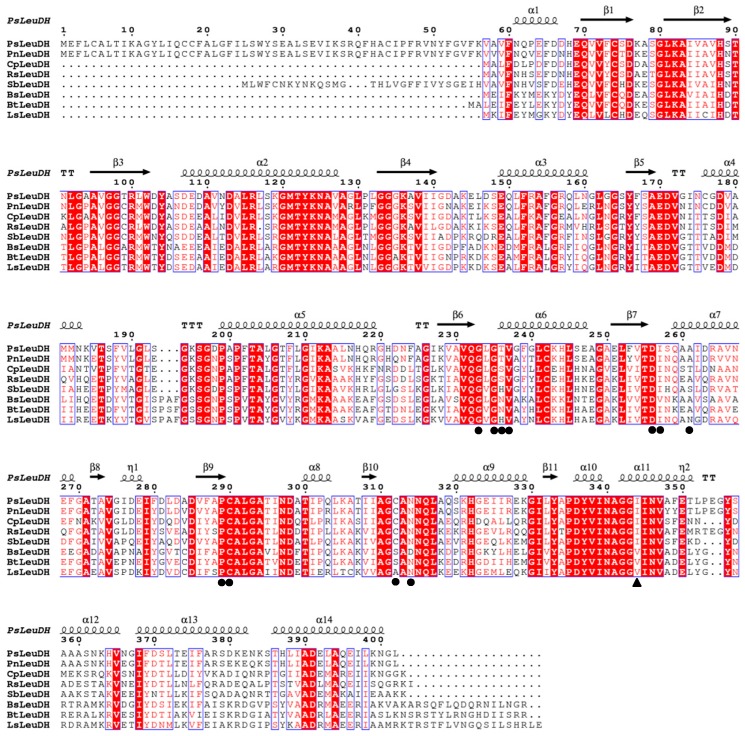
Amino acid sequence alignment of PsLeuDH and related LeuDH. PsLeuDH, *Pseudoalteromonas* sp. ANT178 LeuDH (MH322031); PnLeuDH, *Pseudoalteromonas nigrifaciens* (ASM53600); *Cp*LeuDH, *Colwellia piezophila* (WP_019029130); RsLeuDH, *Rheinheimera salexigens* (WP_070050751); SbLeuDH, *Shewanella baltica* BA175 (AEG11165); BsLeuDH, *Bacillus sphaericus* ATCC4525 (PDB ID:1LEH); BtLeuDH, *Bacillus thuringiensis* (WP_001162678); and LsLeuDH, *Laceyella sacchari* (KR065697). ●, NAD binding site; ▲, Phe binding site.

The *psleudh* gene was amplified from genomic DNA of the strain ANT178. It consisted of an ORF of 1209 bp, encoded a protein of 402 amino acid resides with a theoretical p*I* of 5.08. Furthermore, the DNA sequence of *psleudh* was submitted to the GenBank database with the accession number of MH322031. Based on sequences alignment, PsLeuDH showed the highest sequence similarity (88.0%) with LeuDH from *Pseudoalteromonas nigrifaciens* (ASM53600), followed by a sequence similarity of 65.0% with LeuDH from *Colwellia piezophila* (WP_019029130). More importantly, PsLeuDH had a conserved Phe binding site (I344) and NAD^+^ binding sites (G233, G235, T236, V237, D256, I257, A261, C290, A291, C312, and N314). The coenzyme binding domain of NAD^+^ in LeuDH was capable of catalyzing the reversible oxidative deamination of l-leucine and several other branched chain amino acids to form the corresponding 2-oxo acid derivatives. This domain could be classified as a member of the Rossmann fold superfamily, comprising a plurality of different dehydrogenases, wherein the amino acid dehydrogenase family comprises a common feature: a beta-sheet-alpha helix-beta sheet conformation [15]. PsLeuDH had this structural feature from Figure 1, further demonstrating that PsLeuDH was a member of the Glu/Leu/Phe/Val dehydrogenase family.

### 2.2. Homology Modeling and Analysis of PsLeuDH

**Figure 2:**
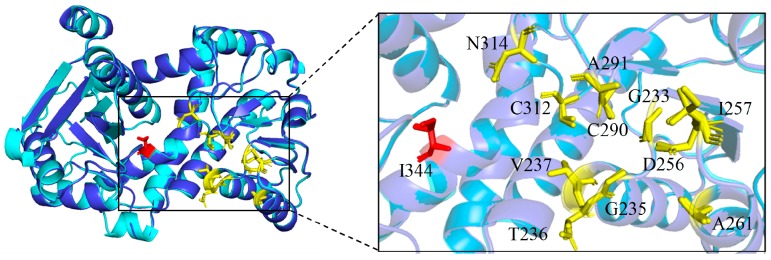
Three-dimensional structure comparison of PsLeuDH and BsLeuDH model. PsLeuDH, tv-blue; BsLeuDH, cyan; NAD^+^ binding site, yellow ball stick model; Phe binding site, red ball stick model.

BsLeuDH (PDB ID:1LEH), encoded 364 amino acids, was isolated from mesophilic bacteria *Bacillus sphaericus* ATCC4525 [16], which exhibited the highest sequence identity (51%) to PsLeuDH using DALI server. The comparative analysis of the 3D structure of PsLeuDH and the mesophilic enzyme Bs-LeuDH was shown in Figure 2. It could be seen that two LeuDHs had a similar NAD^+^ binding site and Phe binding site.

Comparison of structural adaptation characteristics and amino acid substitutions between PsLeuDH and BsLeuDH was shown in Table 1. It can be seen that PsLeuDH exhibited several cold-adapted features. Firstly, the number of electrostatic interactions of PsLeuDH was less than BsLeuDH, which might make the structure of PsLeuDH more flexible [17]. PsLeuDH also had less hydrophobic interactions compared to BsLeuDH, it might make PsLeuDH less rigid and contributed to decrease in structural stability [18]. Secondly, PsLeuDH revealed higher glycine residues and fewer proline and arginine residues that could affect the cold-adapted proteins properties which might offer higher flexibility to proteins [19]. Several amino acid residues in BsLeuDH were replaced by glycine residues in PsLeuDH. The glycine residues might improve the flexibility of the active site, and regulate the entropy of protein unfolding [10], thus probably improving the catalytic efficiency of the enzyme at low temperature. Additionally, proline might reduce the configuration entropy of the unfolding of protein molecules [20] and reduce the stability of enzyme molecules. Additionally, the stability of enzyme was also a significant factor to determine its catalytic characteristics. Some arginine residues in PsLeuDH were replaced by other residues at the same position in BsLeuDH. One of the stability factors in protein structure referred to salt bridges formed by arginine residues [19], arginine might make protein molecules more stable through ionic interaction. Compared with mesophilic enzyme BsLeuDH, PsLeuDH had higher flexibility and lower thermal stability, resulting in higher catalytic efficiency at low temperature [21].

### 2.3. Expression and Purification of the rPsLeuDH

**Figure 3:**
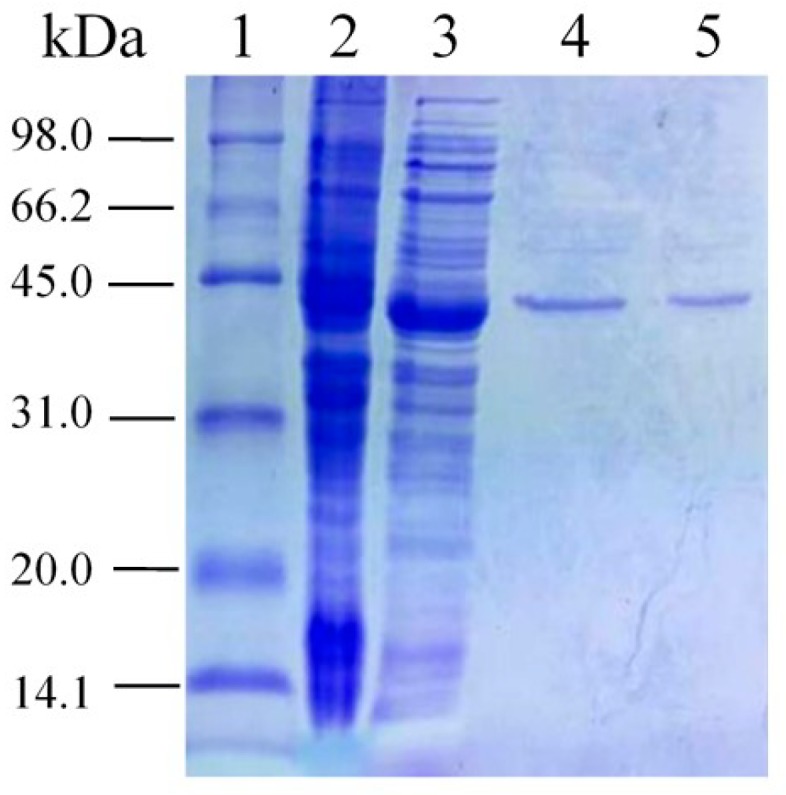
Expression and purification analysis of PsLeuDH. Lane 1: molecular weight standard marker; Lane 2: crude extract from the BL21/pET-28a (+); Lane 3: crude extract from the BL21/pET-28a (+)-PsLeuDH with IPTG induction; Lane 4: rPsLeuDH eluted with 50 mM imidazole; Lane 5: rPsLeuDH eluted with 100 mM imidazole.

The gene coding for the PsLeuDH was cloned into the pET-28a (+) vector and expressed in *E. coli* BL21 (DE3) under IPTG induction (Figure 3, Lane 3). rPsLeuDH was purified in a single step using His-tag affinity chromatography. A major band was observed on SDS-PAGE with about the molecular weight 44.4 kDa (Figure 3, Lane 4, 5). It is noteworthy that the last purified rPsLeuDH exhibited the highest specific activity of 275.13 U/mg.

### 2.4. Effects of Temperature and pH on Activity and Stability of rPsLeuDH

**Figure 4:**
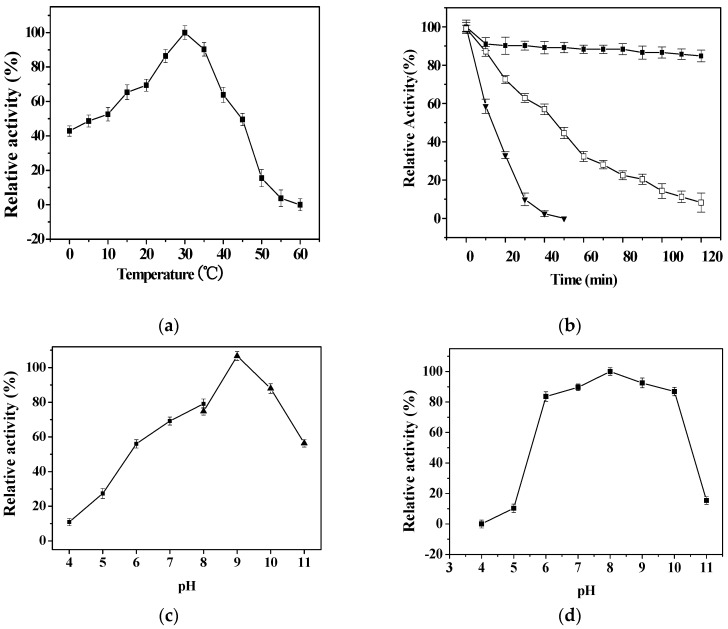
Effects of temperature and pH on the activity and stability of rPsLeuDH. (**a**) Effect of temperature on the activity of rPsLeuDH. (**b**) Effect of temperature on the stability of rPsLeuDH. (■) 30 °C, (□) 40 °C, (▼) 50 °C. (**c**) Effect of pH on the activity of rPsLeuDH. (**d**) Effect of pH on the stability of rPsLeuDH. Data are presented as mean ± SD (*n* = 3).

The temperature characteristic of rPsLeuDH was shown in Figure 4a. It exhibited the highest activity at 30 °C, and that of a cold-adapted LeuDH was 30 °C [12], whereas thermophilic LeuDH was approximately 40–65 °C [6,22], or (60–75 °C) [5]. It is worth pointing out that rPsLeuDH retained 40% of the highest activity at 0 °C, suggested that the enzyme is a cold-adapted enzyme [23]. Furthermore, the thermostability of rPsLeuDH was assessed in Figure 4b. It was stable and retained 85% of its initial activity after incubating at 30 °C after 120 min. While, after incubating at 50 °C for 20 min, it was only 30% of its activity lower than other cold-adapted LeuDHs from *Alcanivorax dieselolei* [12] and *Sporosarcina psychrophila* [7]. However, thermostable LeuDH could retain full activity after incubation at 65 °C for 10 min [24]. The above results indicated that rPsLeuDH had thermal instability, which was another significant feature of cold-adapted enzyme [25]. The effect of pH on rPsLeuDH activity was shown in Figure 4c. The activity of rPsLeuDH was higher under alkaline conditions (pH 7.0–10.0), with the highest activity at pH 9.0. Similar results were described in other LeuDHs such as *Sporosarcina psychrophile* (pH 8.5–11.0) [7], *Laceyella sacchari* (pH 9.5–11) [6] and *Citrobacter freundii* (pH 9.0 to 11.0) [5]. After 30 min of exposure to pH 6.0–10.0, the stability of rPsLeuDH showed a similar pattern with that of the activity response to pH (Figure 4d). This broad range of pH dependence for the activity and stability made the rPsLeuDH probably useful for medical industrial applications.

### 2.5. Effects of NaCl Concentration and Different Reagents on the Activity of PsLeuDH

**Figure 5:**
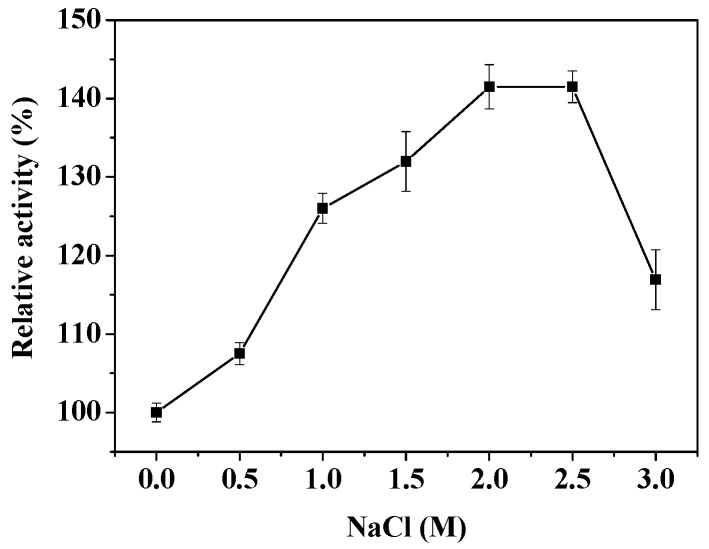
Effect of salt concentration on the activity of rPsLeuDH.

The effect of NaCl concentration on the rPsLeuDH activity was shown in Figure 5. It could be seen that rPsLeuDH was stable at 0–3.0 M NaCl, with the highest activity at 2.0 M NaCl, which may be related to high salinity in the Antarctic sea ice environment. The similar result was also found in LeuDH from *Bacillus licheniformis* [3] and *Thermoactinomyces intermedius* [24] after high salt concentration treatment. The effect of various reagents on the rPsLeuDH activity was listed in Table 2. rPsLeuDH was completely inhibited by 1 mM Pb(NO_3_)_2_ and BaCl_2_. Inhibitions by 1 mM CrCl_2_ and CdCl_2_ were 86.7% and 92.4%, respectively, while only partially inhibited by other metals salt in some extent. In addition, rPsLeuDH was sensitive to Thiourea and ethanol, but Triton X-100 kept the enzyme activity.

### 2.6. The Substrate Specificity Analysis and Kinetic Parameters of rPsLeuDH

The substrate specificity analysis of rPsLeuDH was listed in Table 3. It could catalyze and utilize five substrates, indicating that rPsLeuDH possessed a broad spectrum of substrates in catalytic oxidation reaction. l-leucine was the most suitable substrate for rPsLeuDH, which was the similar with other microbial LeuDH [6,22]. The kinetic parameters of rPsLeuDH were determined. *K*_m_ and *V*_m_ of l-leucine were calculated as 0.33 mM and 15.24 μmol/min·mg, respectively. Besides, the *k*_cat_ value of l-leucine was 30.13/s, demonstrating that rPsLeuDH had a high affinity to substrates and was conducive to improving catalytic efficiency at low temperature.

### 2.7. The Thermodynamic Parameters of rPsLeuDH

Thermodynamic parameters such as Δ*H*, Δ*S* and Δ*G* at different temperature (0–30 °C) were calculated and listed in Table 4. At 0, 10, 20, and 30 °C, the *k*_cat_ value of rPsLeuDH were 12.25, 14.96, 20.20 and 30.13/s, respectively, indicating that the *k*_cat_ value increased with increasing temperature, which was similar to the *k*_cat_ change trend of cold-adapted β-d-galactosidase at different temperatures [26]. rPsLeuDH also exhibited lower Δ*H*, Δ*S* and Δ*G* and higher *k*_cat_ at low temperature, as compared to mesophilic enzyme, which may be mainly related to the conformation of cold adapted protein [27]. On the other hand, it may also be related to increasing the efficiency of binding of the substrate to the catalytic site [28].

## 3. Materials and Methods

### 3.1. Microorganisms and Growth Conditions

The strain *Pseudoalteromonas* sp. ANT178, isolated from sea ice in Antarctica (68°30′ E, 65°00′ S), was used as a source of *psleudh* gene. The strain ANT178 was cultivated in the 2216E sea water medium (initial pH 7.5, 5 g/L peptone, and 1 g/L yeast extract) for 96 h at 12 °C. *E. coli* BL21 (DE3) was used as the plasmid host.

### 3.2. Sequence Analysis of LeuDH Gene

The open reading frame and amino acid sequences of *psleudh* gene were computed (https://www.ncbi.nlm.nih.gov/orffinder/). The theoretical molecular weight and p*I* were also analyzed using the ExPASy Compute p*I*/Mw tool (http://web.expasy.org/computepi). Multiple sequence alignment of the amino acids of PsLeuDH was performed using Bioedit 7.2 and ESPript 3.0 [29].

### 3.3. Protein Homology Modeling

A homology model of LeuDH was built with SWISS-MODEL. LeuDH from mesophilic bacteria *Bacillus sphaericus* ATCC4525 (PDB ID:1LEH) [16] was selected as the template. The structure figures were created with PyMOL software (DeLano Scientific LLC, San Carlos, CA, USA). Salt bridges were carried out using VMD 1.9.3. (University of lllinois Urbana-Champaign, Champaign, IL, USA). For the hydrogen bonds, a cut-off distance of 3.3 Å was set. Cation-pi interactions, aromatic interactions, ionic interactions, and hydrogen bonds were predicted by the Protein Interactions Calculator program (http://pic.mbu.iisc.ernet.in).

### 3.4. Molecular Cloning, Expression and Purification of rPsLeuDH

The genome of *Pseudoalteromonas* sp. ANT178 was sequenced and annotated using high-throughput technologies (data not shown). The full-length gene of *psleudh* was amplified by PCR using the primers 5′-GATGGATCCATGGAATTT TTATGTG-3′ (*Bam*HI site underlined) and 5′-CAGAAGCTTGAAGACCGTTTT TAAG-3′ (*Hin*dIII site underlined) according to its genome sequence. PCR was performed with Taq DNA polymerase (TaKaRa Bio, Dalian, China). The product was then directly cloned into the corresponding sites of the pET-28a (+) vector and transformed into *E. coli* BL21. The transformants with the *psleudh* gene were grown in Luria-Bertani (LB) medium supplemented with 100 mg/L kanamycin and cultured by shaking at 37 °C until the OD_600_ reached 0.6–0.8. Then, 1.0 mM sopropyl-β-d-thiogalactopyranoside (IPTG) was added for induction. The bacterial cells were cultured at 37 °C for 2–3 h, and then the culture temperature was shifted to 28 °C to induce the protein expression for 6 h. The induced cells centrifuged at 4 °C and 7500× *g* for 15 min and subjected to ultrasonic disruption with 150 W (JY96-IIN, Shanghai, China). The insoluble debris was removed by centrifuged at 4 °C and 7500× *g* for 15 min, and the supernatant was harvested as crude protein (21.99 mg). Purification of rPsLeuDH with the His-tagged was purified using Ni-NTA affinity chromatography. The purified protein (1.11 mg) was eluted with 10, 50, 100 and 250 mM imidazole buffer (20 mM Tris-HCl, 500 mM NaCl, pH 8.0) at a flow rate of 1.0 mL/min. The purity and the molecular mass of the rPsLeuDH were determined by SDS-PAGE, using 12.0% polyacrylamide gels.

### 3.5. Assay of rPsLeuDH Activity

The standard enzyme assay were based on traditional method and modified on basis [1,30]. The oxidation reaction activity assay was determined by 200 μL reaction system. It contained 0.1 M Glycine-NaOH (pH 10.4) buffer, 10 mM l-leucine and 10 μL purified enzyme (0.62 μg), which incubated at 30 °C for 2 min. After adding 1 mM NAD^+^, the changes of absorbance at 340 nm within 1 min were detected. Futhermore, the reductive amination reaction system containing (200 μL) 0.2 M NH_4_Cl-NH_4_OH buffer (pH 9.0), 5 mM TMP and 10 μL purified enzyme at 30 °C for 2 min, after adding 0.2 mM NADH, changes in absorbance at 340 nm within 1 min were measured. One unit of LeuDH activity was defined as the amount of enzyme catalyzed the formation or reduction of 1 μmoL NADH/min at 30 °C.

### 3.6. Characterization of the Purified rPsLeuDH

The optimal temperature of the purified rPsLeuDH was determined with the standard assay at temperatures from 0 °C to 60 °C. To evaluate the thermostability, the purified enzyme was incubated at three different temperatures (30, 40, and 50 °C) for 120 min, and the residual activity was measured by the standard enzyme assays. The optimal pH of the purified enzyme was determined at 30 °C using Citric acid/Na_2_HPO_4_ buffer (0.2 M) and NH_4_Cl-NH_4_OH buffer (0.2 M) for pH ranges 4.0–8.0 and 8.0–10.0, respectively. To assess pH stability, the rPsLeuDH was pretreated at pH 4.0–11.0 in the absence of substrate at 30 °C for 30 min, and the residual activity was measured by the standard enzyme assays. The purified rPsLeuDH was incubated at 0–3.0 M NaCl at 30 °C for 30 min, and remaining activity was assayed with the standard enzyme assays. The effects of different reagents on the rPsLeuDH activity were assayed with the standard enzyme assay after pre-incubating enzyme in different metal ions at 30 °C for 30 min. Enzyme activity assayed without any reagent was defined as control (100%).

### 3.7. Kinetic Parameter of the rPsLeuDH

To assess the kinetics parameters, the Lineweaver-Burk plot method was used to calculate the *K*_m_ and *V*_m_ of rPsLeuDH [31]. The kinetic constants of NADH (0.025 mM–0.4 mM), l-leucine (0.05 mM–2 mM), l-tyrosine (0.05 mM–2 mM), l-proline (0.05 mM–2 mM), dl-methionine (0.05 mM–2 mM), l-arginine (0.05 mM–2 mM), TMP (0.05 mM–2 mM), and NAD^+^ (0.025 mM–0.4 mM) were determined by the above method in rPsLeuDH.

### 3.8. Thermodynamic Parameter of the rPsLeuDH

The *k*_cat_ parameter is the reaction rate constant for the enzymatic-substrate complex chemical conversion into the enzyme and the product. *k*_cat_ was calculated based on kinetics experiments, and the thermodynamic related parameters were assayed by the modification method of Feller [27] as follows:

(1) $$k_{\mathrm{cat}}=Ae^{\frac{-E_{a}}{RT}}$$

(2) $$∆H=E_{a}-RT$$

(3) $$∆S=R\left(Ink_{\mathrm{cat}}-24.76-InT+\frac{E_{a}}{RT}\right)$$

(4) ∆G=∆H−T∆S

where *A* is the constant, *E*_a_ is the activation energy of the reaction, *R* is the gas constant (8.314 J mol^−1^ K^−1^), ∆*H* is the enthalpy of activation, ∆*S* is the entropy of activation, and ∆*G* is the free energy of activation.

## 4. Conclusions

A novel cold-adapted leucine dehydrogenase gene (*psleudh*) was cloned from Antarctic sea-ice bacterium and expressed in *E. coli* (DE3). Through homology modeling and comparison with its homologous enzyme (BsLeuDH), it was suggested that more glycine residues, reduced proline residues and arginine residues might be responsible for its catalytic efficiency at low temperature. rPsLeuDH was purified and characterized with higher activity at 30 °C, high salt (3.0 M), remarkable pH stability (pH 6.0–10.0), and higher specific activity (275.13 U/mg). These unique properties of rPsLeuDH make it a promising candidate as a biocatalyst in the enzymatic production of l-*tert*-leucine at room temperature.

## Figures and Tables

**Table 1:** Comparison of structural adaption features and amino acid substitutions between PsLeuDH and its homolog (BsLeuDH).

Parameters	PsLeuDH	Bs-LeuDH	Expected Effect on PsLeuDH
Electrostatic interactions			Protein stability
Salt Bridge (2.5 to 4.0)	17	22	
Hydrogen Bonds (≤3.3 Å)	368	403	
Cation-pi interactions	3	11	
Aromatic interactions	6	8	
Hydrophobic interactions	227	318	Thermolability
Glycine residues	42	36	Flexibility
Proline residues	9	11	
Arginine residues	10	17	
Glycine substitution (PsLeuDH → BsLeuDH)	G163 → N107, G177 → D121, G238 → A185, G240 → A187, G275 → A222, G401 →V346		
Proline substitution (PsLeuDH → BsLeuDH)	A94 → P38, A143 →P87, S320 → P267, S385 → P330		
Proline substitution (PsLeuDH → BsLeuDH)	P131 → N75, P63 → M7		Stability
Arginine substitution (PsLeuDH → BsLeuDH)	R219 → F166, R264 → A211, R327 → H274, R378 → I323		

**Table 2:** Effects of different reagents on the activity of rPsLeuDH.

Reagent	Concentration	Relative Activity (%)	Reagent	Concentration	Relative Activity (%)
None		100 ± 0.0			
KCl	1 mM	99.7 ± 1.6	KCl	5 mM	40.0 ± 1.9
CoCl_2_	1 mM	90.1 ± 1.7	CoCl_2_	5 mM	70.0 ± 2.0
MgCl_2_	1 mM	87.9 ± 0.8	MgCl_2_	5 mM	65.8 ± 1.2
CaCl_2_	1 mM	87.9 ± 0.4	CaCl_2_	5 mM	68.1 ± 0.9
ZnCl_2_	1 mM	80.0 ± 2.5	ZnCl_2_	5 mM	72.2 ± 2.0
FeCl_2_	1 mM	75.1 ± 2.2	FeCl_2_	5 mM	62.4 ± 1.7
CuCl_2_	1 mM	61.0 ± 2.2	CuCl_2_	5 mM	41.0 ± 1.5
HgCl_2_	1 mM	29.2 ± 0.3	HgCl_2_	5 mM	12.3 ± 1.9
CrCl_2_	1 mM	13.3 ± 0.3	CrCl_2_	5 mM	5.8 ± 2.9
CdCl_2_	1 mM	7.6 ± 0.5	CdCl_2_	5 mM	0.0 ± 0.0
Pb(NO_3_)_2_	1 mM	0.0 ± 0.0	Pb(NO_3_)_2_	5 mM	0.0 ± 0.0
BaCl_2_	1 mM	0.0 ± 0.0	BaCl_2_	5 mM	0.0 ± 0.0
EDTA	1 mM	91.8 ± 2.7	EDTA	5 mM	84.2 ± 2.1
Thiourea	1 mM	51.5 ± 4.0	Thiourea	5 mM	34.3 ± 2.6
Triton X-100	0.2%	102.7 ± 1.4	Ethanol	25%	67.5 ± 1.4

**Table 3:** Substrate specificity analysis of rPsLeuDH.

Substrate	*V*_m_ (μmol/min·mg)	*K*_m_ (mM)	*k*_cat_ (1/s)	*k*_cat_/*K*_m_ (mM^−1^ s^−1^)
l-lecine	15.24	0.33	30.13	91.30
l-tyrosine	13.35	0.48	26.39	54.98
l-proline	10.52	0.64	20.80	32.50
dl-methionine	8.38	0.75	16.57	22.09
l-arginine	7.13	0.84	14.09	16.77

**Table 4:** Thermodynamic parameter of the rPsLeuDH.

Temperature (°C)	Δ*H* (KJ/mol)	Δ*S* (J/mol K)	Δ*G* (KJ/mol)	*k*_cat_ (1/s)
0	18.27	−156.45	61.01	12.25
10	18.19	−157.75	62.90	14.96
20	18.11	−158.02	64.43	20.20
30	18.02	−157.28	65.70	30.13
